# Effect of excessive CO_2_ on physiological functions in coastal diatom

**DOI:** 10.1038/srep21694

**Published:** 2016-02-15

**Authors:** Feng-Jiao Liu, Shun-Xing Li, Bang-Qin Huang, Feng-Ying Zheng, Xu-Guang Huang

**Affiliations:** 1 Fujian Province Key Laboratory of Modern Analytical Science and Separation Technology, Minnan Normal University, Zhangzhou, China, 363000; 2 College of the Environment & Ecology, Xiamen University, 361005, China

## Abstract

Rising dissolution of anthropogenic CO_2_ in seawater may directly/indirectly cause ocean acidification and desalination. However, little is known about coastal physiological functions sensitivity to these processes. Here we show some links between ocean acidification/desalination and physiological functions in *Thalassiosira weissflogii*. Cell density (CD), protein, chlorophyll *a* (Chl *a*), malonaldehyde (MDA), superoxide dismutase (SOD), and carbonic anhydrase (CAs) were determined for the assessment of algal biomass, nutritional value, photosynthesis and respiration, lipid peroxidation, antioxidant capacity, and carbon sequestration ability. The influence of pH on the algal Chl *a* and MDA were extremely significant (*P* < 0.01). Salinity (S) on cell density and acidity (pH) on protein was significant (0.01 < *P* < 0.05). Additionally, a significant negative-correlation was observed between cell density and CAs. CAs and SOD had negatively correlations with CD, Chl *a*, protein, and MDA under pH or S influence, but positive correlation between themselves. Coastal physiological functions were affected by increasing order was acidification* *< acidification + desalination < desalination for Chl *a* and protein, desalination < acidification + desalination < acidification for SOD and CAs. Thus, the ongoing excessive CO_2_-driven ocean acidification and desalination should be of high attention when assessing the risks of climate change on coastal phytoplankton.

Human induced climate change effects marine environment in the next century and beyond. Future scenarios predict an increasing CO_2_ partial pressure (pCO_2_) in the atmosphere approximately from 380 to 1,000 ppm until the end of this century^1^,^2^,^3^. Approximately a quarter of the anthropogenic CO_2_ is absorbed by the ocean. This oceanic uptake of CO_2_ leads to a change in marine carbonate chemistry resulting in a decrease of seawater pH and carbonate ion concentration, a process commonly called “Ocean Acidification (OA)”^4^. OA is a potential threat to marine ecosystems through its effect on the physiology and ecology of many marine species^5^,^6^,^7^. OA is also likely to have measurable biological consequences on marine biodiversity and ecosystem functioning, as well as on the provision of ecosystem services^8^,^9^,^10^,^11^.

Climate change results in ocean warming, sea ice coverage reducing (or sea ice melt increasing), global sea-level rising, surface evaporation increasing, and patterns of deep-water ventilation changing, potentially altering future surface ocean carbonate conditions and acidification^7^,^12^. Along with warmer air and ocean temperatures, increased sea ice melt and had risen global sea-level can lead to salinity declining^13^. Simultaneously, climate change induced heavy precipitation is expected to reduce surface salinity^14^. Where mixing with fresh water runoff from river mouths, seawater salinity can be substantially decline.

The growth and photosynthesis of some seaweeds and seagrasses will be benefit under OA conditions^15^. Furthermore, OA could interact with other stressors, such as increasing light, warming water, decreasing oxygen concentration, overfishing, and after eutrophication, showing an additive effect on these stressors^8^,^16^. The rising CO_2_ combined with light stress may reduce primary production of phytoplankton^17^. However, the influence of ocean desalination and its coexistence with acidification on coastal physiological functions is unknown.

Coastal ecosystems account for over a third of the world’s ecosystems services, such as nitrogen fixation, phytoplankton abundance and production, bacteria growth rates, food-wed type, and the interactions between animals, plants and bacteria^18^,^19^,^20^,^21^,^22^. Diatoms are the dominant group of phytoplankton in the modern ocean, specially in well-mixed coastal upwelling regions^23^,^24^, accounting for approximately 40% of oceanic primary productivity and critical foundation of coastal food web^25^,^26^. Coastal physiological functions (including cell density (CD), protein, chlorophyll a (Chl *a*), malonaldehyde (MDA), superoxide dismutase (SOD), and carbonic anhydrase (CAs)) have been used in our laboratory for the estimation of algal biomass, nutritional value, photosynthesis and respiration, lipid peroxidation, antioxidant capacity, and carbon sequestration ability, respectively. Algal density controls the stability of food chains and the energy transfer in the food chains of marine systems^27^. Protein synthesis is one of nitrogen assimilations and its concentration is an indicator of metabolic capacity and nutritional value^28^. Chlorophyll *a* is an important material for algal photosynthesis and respiration, and its concentration is an important indicator for estimating algal primary productivity^28^. SOD is one kind of metalloenzyme that can catalyze the dismutation process of superoxide anion into oxygen and hydrogen peroxide, Thus SOD provides protection for the survival of aerobic organisms^29^,^30^. MDA, a product of lipid peroxidation, can be used as an indicator of free radical activity and tissue damage^31^. CAs is a zinc-containing metalloenzyme that catalyzes the interconversion of CO_2_ and HCO_3_^−^ in many organisms, has been taken as a component of carbon-concentrating mechanisms and plays a role in photosynthetic CO_2_ fixation^32^,^33^.

## Results

### Influence of S or/and pH on Cell Density

**Figure 1:**
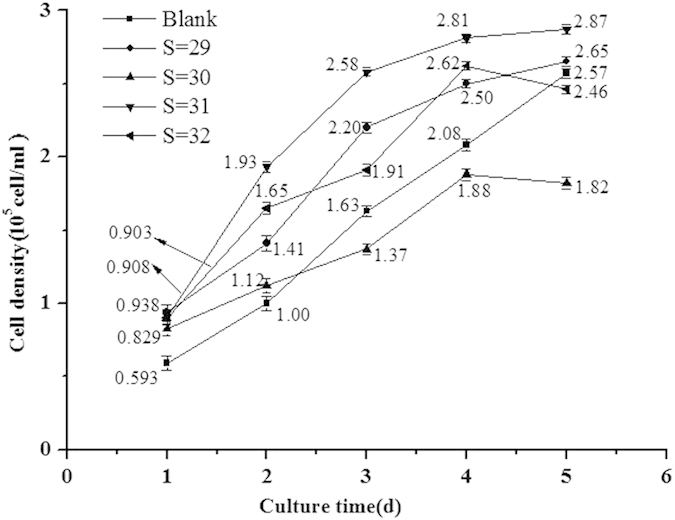
Influence of salinity (S) on algal density. (a) S = Blank; (b) S = 29; (c) S = 30; (d) S = 31; (a) S = 32. Data are mean ± SD (*n* = 3).

**Figure 2:**
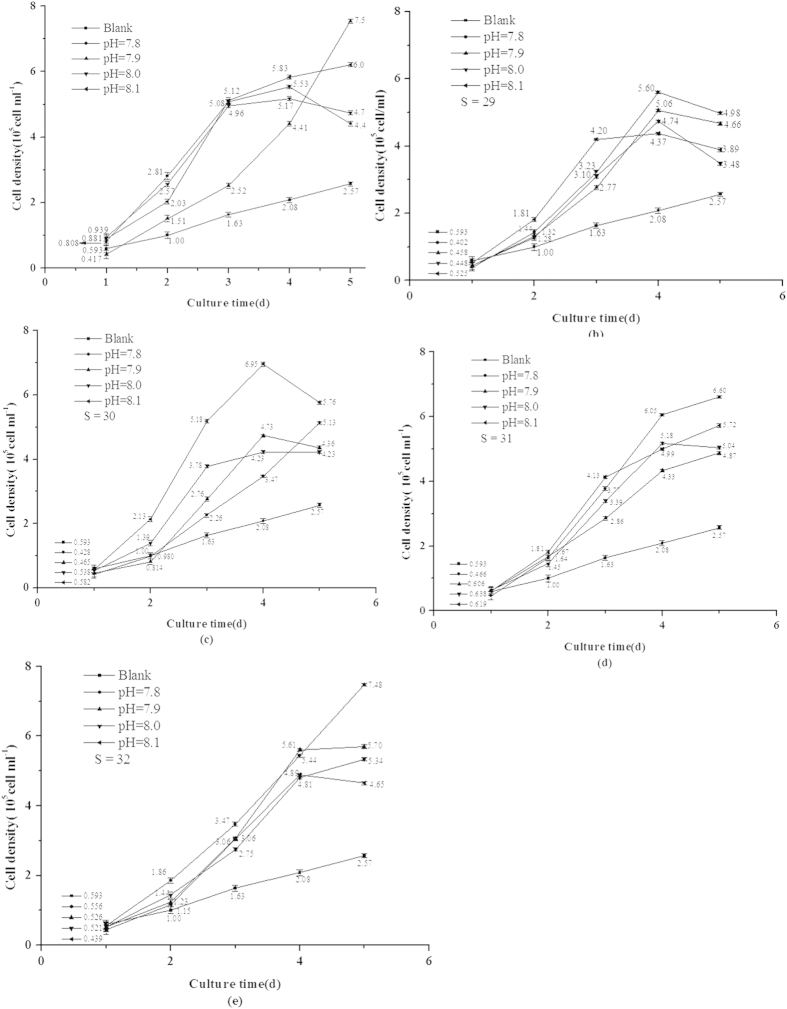
Influence of salinity (S) and acidity (pH) on algal density. Data are mean ± SD (*n* = 3).

Cell growth rate was controlled by the speed of cell division. The results were shown in Figs 1 and 2 indicated that cell division speed could be inhibited by desalination but promoted by acidification. When coexistence with S and pH, cell division could be promoted, i.e., cell densities were all higher than blank experiment, but cell growth cycles were shorten significantly, the results were shown in Fig 2. When pH was 7.8 and S was decreased from 32 to 30, cell growth was increased continuously, but cell density was decreased. The overall observed changes was acidification < acidification + desalination < desalination.

### Influence of S or/and pH on Phytoplankton Physiological Properties

**Figure 3:**
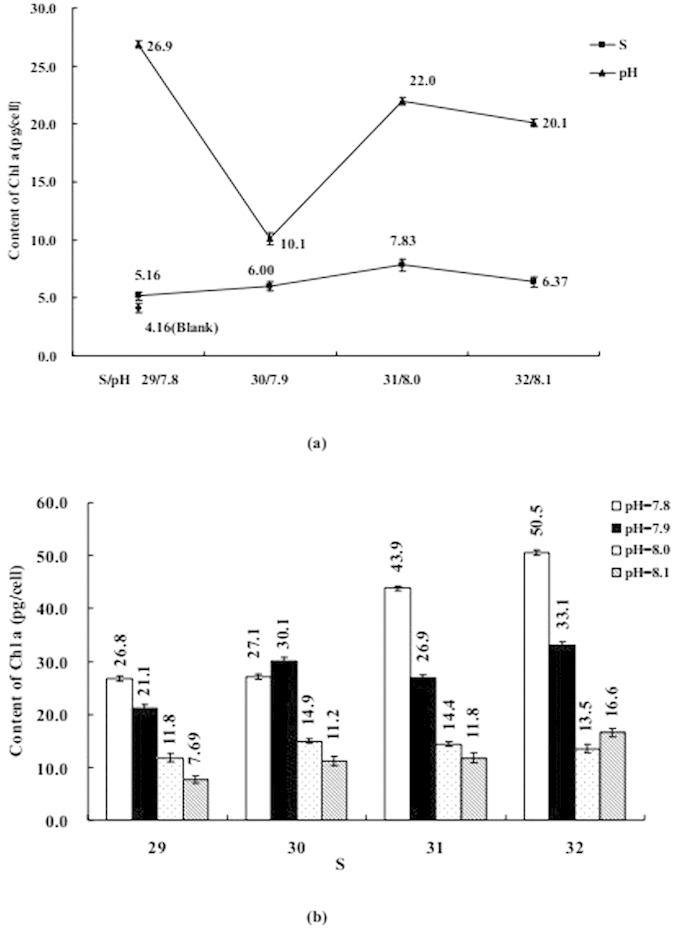
(**a**) Influence of salinity (S) or acidity (pH) on the content of chlorophyll a (Chl *a*); (**b**) Influence of S and pH on the content of Chl *a*. Data are mean ± SD (n = 3).

**Figure 4:**
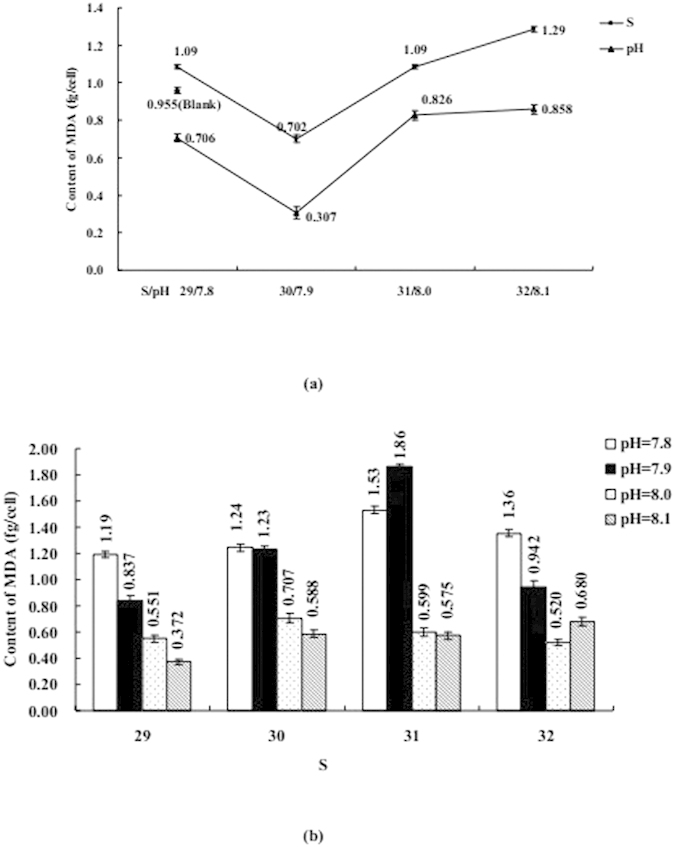
(**a**) Influence of salinity (S) or acidity (pH) on the content of malonaldehyde (MDA); (**b**) Influence of S and pH on the content of MDA. Data are mean ± SD (n = 3).

**Figure 5:**
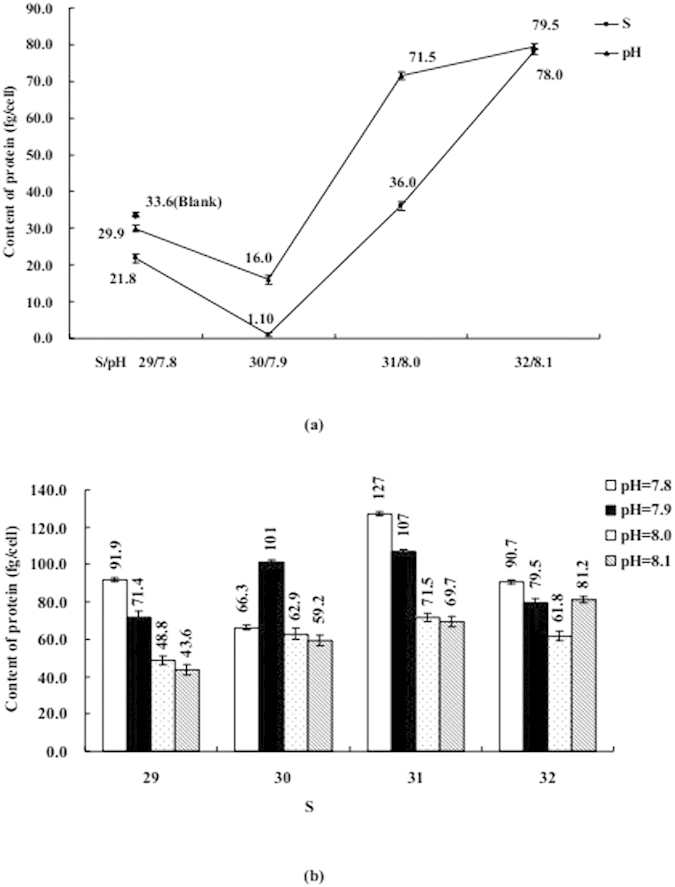
(**a**) Influence of salinity (S) or acidity (pH) on the content of protein; (**b**) Influence of S and pH on the content of protein. Data are mean ± SD (n = 3).

**Figure 6:**
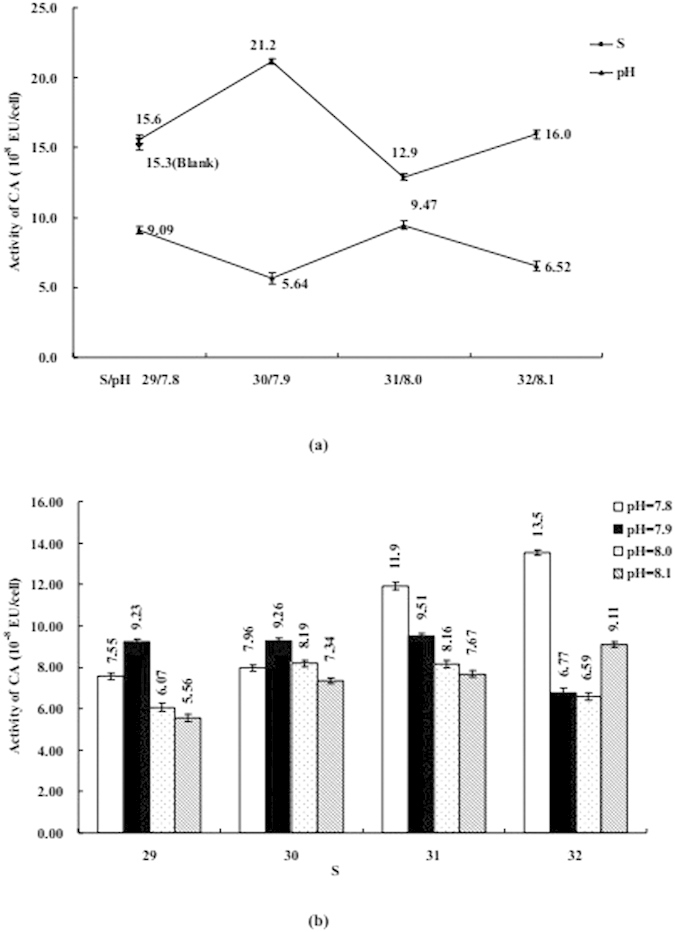
(**a**) Influence of salinity (S) or acidity (pH) on the activity of carbonic anhydrase (CA); (**b**) Influence of S and pH on the activity of CA. Data are mean ± SD (n = 3).

**Figure 7:**
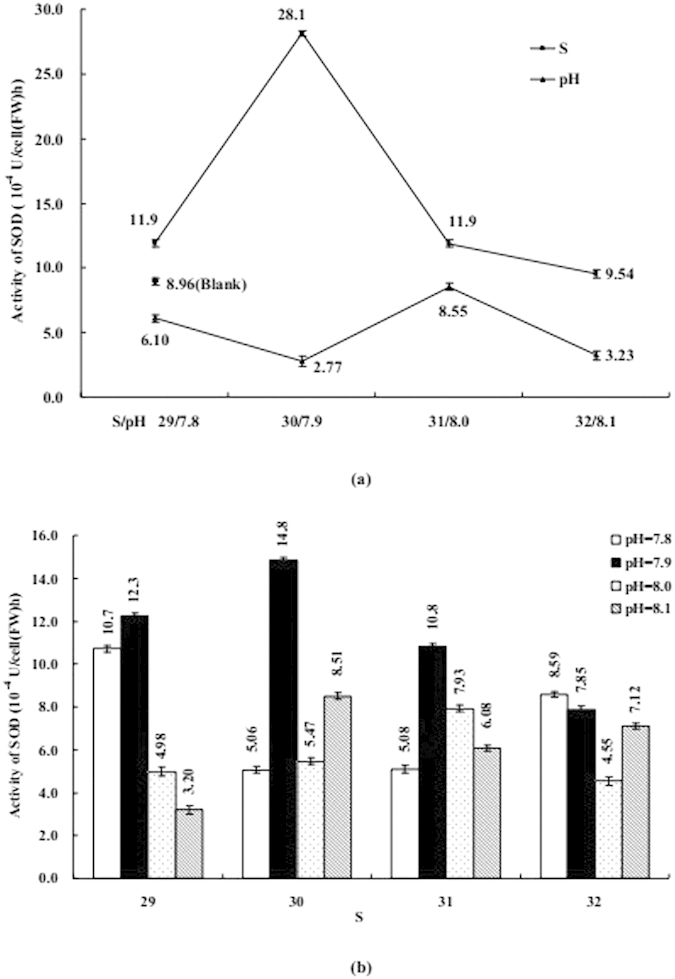
(**a**) Influence of salinity (S) or acidity (pH) on the activity of superoxide dismutase (SOD); (**b**) Influence of S and pH on the activity of SOD. Data are mean ± SD (n = 3).

The influence of S or/and pH on coastal diatom can be seen in Figs 3, 4, 5, 6, 7, including Chl *a*, protein, MDA, SOD, and CAs. Figures 3, 4, 5 mainly display the effect of S on concentration of Chl *a*, protein, and MDA was clearly obvious when compared with pH. However, the influence trends were reversed as SOD and CAs were observed to be low. The variation trend of protein and MDA affected by S and pH was similar, but that was reverse to CA activity; Influence trend of pH on Chl *a*, SOD, and CAs was also similar.

Coexistenced with S and pH, the concentrations of Chl *a* and activities of CAs were decreased with desalination when pH was 7.8 and 8.1, protein was similar when S from 32 to 29 and pH was 8.1. At the same time, the concentrations of MDA and protein and activities of CAs were decreased with S decreased from 31–29 when pH was 7.9, but the influence trends were reversed while S was over the above mentioned range. When pH was 7.9 and 8.0 the influence of desalination on SOD activity was similar.

The influence degree on coastal physiological functions was different, acidification < acidification + desalination < desalination for Chl *a* and protein, desalination < ccidification + desalination < acidification for SOD and CAs.

There were significant relationships in the six tested parameters, although these were not always consistent (Table S1). There was a very significant negatively correlations between CD and CAs (r = −0.996, *P *< 0.01, S; r = −0.966, *P *< 0.05, pH). CAs and SOD had negatively correlations with CD, Chl *a*, protein, and MDA under pH or S influence, but positive correlation between themselves (r = 0.950, pH; r = 879, S). Between protein and MDA was also positively correlated (r = 0.900, S; r = 0.870, pH).

These data were much higher than their corresponding detection limit and within their linear range. The varying of these data could be accepted, shown in Figs 1, 2, 3, 4, 5, 6, 7. According to ANOVA analysis as shown in Table S2, the influence of pH on the algal Chl *a* and MDA were extremely significant (*P *< 0.01, F > F*α*), S on CD and pH on protein were significant (0.01 < *P *< 0.05, F > F*α*).

## Discussion

Cell growth rate is controlled by the speed of cell division and directly affected by ocean acidification^34^. Similar to previously published results^35^,^36^, our study concords with these observations, showing a that algal density decreased with an increase of acidify (e.g. by decreasing pH). Rising atmospheric CO_2_ could increase dissolved CO_2_ concentration in seawater, a moderate increase in CO_2_ facilitates photosynthetic carbon fixation of some phytoplankton groups^37^. However, when the acidity is lower than a certain concentration, it will damage cellular structure and physiological functions^34^. The reduced salinity can cause further osmotic challenge and elevate basal energy demand in a decreased pH environment, leading to lower energy levels available for growth or shell formation in marine organisms^38^,^39^. Thus, it can mitigate biological effects for microalgae. High concentrations of inorganic anions and cations from highly saline environments can potentially reduce the permeability of ionic liquid cations through algal cell walls^40^. Therefore, excessive concentrations of salinity can have a harmful effect on algal cell growth. Our results were agreed well with the results by Stedmon *et al.*^41^.

As previously reported that the synthesis process of protein and Chl *a* is controlled by the activity of NR, GS, G-tRNAS, and NADPH^27^,^28^. Ocean acidification may enhance enzyme activity, including NR, GS, G-tRNAS, and NADPH^42^, and then promote the synthesis of protein (including the other enzymes) and chlorophyll a. When pH was more than a certain concentration, algal cell could induce the overload of hydroxyl radicals, and then inhibited the activities of NR, GS, glutamate-tRNA synthase, and NADPH. Therefore, the influence trends of pH on the content of protein and chlorophyll a were similar and significant (*P *< 0.05, F > F*α*). Our results were in good agreement with the reported influence trends^42^.

SOD activity and MDA content would be changed with the amount of hydroxyl radical (one kind of reactive oxygen species). According to our results, both S and pH could induce the oxidant stress in algal cells. With desalination and acidification, MDA content of algal cells increased, indicating that membrane lipid peroxidation was enhanced. The change in SOD activity of algal cells also confirms the generation of oxidant stress.

CAs abundant enzymes play a key role in carbon-concentrating mechanism of marine phytoplankton. The synthesis process is controlled by an essential element (Zn) in phytoplankton, because of Zn is used as a cofactor in CAs^43^. The decrease in pH will result in reducing the concentrations of hydroxide (OH^−^) in most natural surface waters. Ocean desalination also will reduce the concentration of anion, such as Cl^−^ and OH^−^. When pH in the range from 7.8 to 8.1 and S from 29 to 32, the speciation of Zn included ZnCO_3_, Zn(CO_3_)_2_^2−^, Zn(OH)^+^, ZnCl^+^, Zn^2+^, ZnClOH, ZnCl_2_, and ZnHCO_3_^+^, only a few fraction of Zn^2+^ could be existed. Thus, S and pH could inhibit CAs activity through affecting the availability of Zn. CAs expression is very sensitive to variations in pH–*P*_CO2_^44^,^45^,^46^. Previous studies have also shown a substantial decrease in CAs activity in the diatoms *T. weissflogii* when pH decreases^43^.

Coastal physiological functions could be affected seriously by the carbon dioxide-associated ocean acidification and desalination. Here fully investigated the coexistence of ocean acidification and desalination affect algal biomass, nutritional value, photosynthesis and respiration, lipid peroxidation, antioxidant capacity, and carbon sequestration ability, respectively. At the same time, desalination can be related to the influence of dilute water mouth, and coexistence of acidification and desalination can also be linked to estuary affected by acid rain. However, there has only laboratory tests and should be further investigated and most importantly on scale-up flied experiment for a future angle to risk assessment climate change on coastal environment.

## Methods

### Seawater Sahmple

Seawater was collected from the Taiwan strait (22.65 N, 118.82E) shown in Figure S1 (The R Programming Language, 3.0.3), stored at 4°C for about 6 months, and filtered through 0.22 *μ*m acid-washed capsule filters (Pall Supor membrane, Acropak 200) before use. The salinity (S) and acidity (pH) were measured three times using Salinometer Meter and Delta 320-S pH meter (Mettler-Toledo, Greifensee, Switzerland). The Sal and pH were 32.5 and 8.10, respectively.

### Phytoplankton culture

Unialgal cultures of *T. weissflogi* were obtained from the State Key Laboratory for Marine Environmental Science, Xiamen University. The cultured growth cycle curve was shown in Figure S2. Exponentially growing cells of *T. weissflogii* cells were filtered and transferred to new medium every 1–2 days and then the cells would adapt to the tested experimental conditions. After 4 transfers, the cells (1 × 10^4^ cells mL^−1^) were again filtered and added into 5 L of seawater (filtered with 0.22 *μ*m acid-washed capsule filters) in acid-cleaned polycarbonate bottles. Algal cells were illuminated with a light intensity of 140 *μ*mol photons m^−2^ s^−1^ on a 14 hr: 10 hr light: dark cycle and maintained in seawater at various concentrations of S (29, 30, 31, and 32, respectively) and/or pH/ pCO_2_ (7.8/770 ppm, predicted CO_2_ levels in 2100; 7.9/600 ppm, predicted CO_2_ levels in 2060; 8.0/470 ppm, predicted CO_2_ levels in 2030; and 8.1/380, current CO_2_ levels, respectively)^8^,^47^ at 19 °C. After determination of S and pH in the medium, their values were maintained through compensating addition daily of Na_2_CO_3_ (0.1 mol/L) and CO_2_ for 4 days, respectively, i.e., semi-continuous culture was adopted. Na_2_CO_3_ and CO_2_ were added by drops and gently bubbled with humidified, CO_2_-enriched air, respectively. Marine carbonate system considers CO_2_ (aq) [the sum of CO_2_ and H_2_CO_3_], HCO_3_^−^, CO_3_^2−^, H^+^, OH^− ^48. The additional CO_2_ causes re-equilibration of the seawater carbonate system, increasing the concentrations of aqueous CO_2_ and bicarbonate ion, HCO_3_^−^, while decreasing that of the carbonate ion, CO_3_^2−^. Dissolved inorganic carbon (DIC) is the sum of all dissolved inorganic carbon species, which is the main acid-base buffer of seawater^34^. When addition of Na_2_CO_3_ and CO_2_, the medium was stirring until desirable S and pH were achieved stably. The algal suspensions were stirred at 100 rpm to simulate the current of seawater. Large culture vessels (5 L) were used so as to decrease the thickness of marine phytoplankton suspension to minimize the difference of light illumination between the surface and bottom of marine phytoplankton suspension^49^. These experiments were triplicated (*n* = 3).

### Test methods

Cell density was counted microscopically. SOD activity, and the content of protein, Chl *a*, and MDA was determined by monitoring the inhibition of photochemical reduction of nitroblue tetrazolium chloride, coomassie brilliant blue method, spectrophotometric method, and thiobarbituric acid method, respectively, as described in detail previously^27^. CA activity was measured by an electrometic method as described by Chen and Gao^32^.

### Statistical analysis

Analysis of variance was calculated by using SASPROC MIXED^50^ and *p* value was calculated using two-way ANOVA. The methods of statistical analysis as described in detail previously^28^. *Post hoc* tests were examined using Tukey’s test for two-way ANOVA and Dunnett’s Test for multivariate ANOVA^7^.

## Additional Information

**How to cite this article**: Feng-Jiao, L. *et al.* Effect of excessive CO_2_ on physiological functions in coastal diatom. *Sci. Rep.* **6**, 21694; doi: 10.1038/srep21694 (2016).

## Supplementary Material

Supplementary Information
