# Corrigendum: Effect of excessive CO_2_ on physiological functions in coastal diatom

**DOI:** 10.1038/srep25167

**Published:** 2016-05-09

**Authors:** Feng-Jiao Liu, Shun-Xing Li, Bang-Qin Huang, Feng-Ying Zheng, Xu-Guang Huang

Scientific Reports 6: Article number: 21694;10.1038/srep21694 Published online: 02152016; Updated: 05092016

The original version of this Article incorrectly listed ‘College of Chemistry and Environment, Minnan Normal University, Zhangzhou, China, 363000’ as a present address for Bang-Qin Huang rather than Shun-Xing Li.

This error has now been corrected in the PDF and HTML versions of the Article.

