# Supplementary material for: Effect of excessive CO2 on physiological functions in coastal diatom
Source: Sci Rep. 2016 Feb 15;6:21694. doi: 10.1038/srep21694 (PMC4753682; doi:10.1038/srep21694)
Supplement: Supplementary Information [file srep21694-s1.doc]

**Effect of excessive CO_2_ on physiological functions in coastal diatom**

Liu Feng-Jiao ^a, b^, Li Shun-Xing ^a *^, Huang Bang-Qin^b^, Zheng Feng-Ying ^a^, Huang Xu-Guang ^a^

**Tables and Figure legends**

**Table 1** Correlations between physiological function (i.e., cell density (CD), chlorophyll a (Chl *a*), protein, malonaldehyde (MDA), superoxide dismutase (SOD), and carbonic anhydrase (CA)) under different salinity (Sal) and acidity (pH).

**Table 2.**  Statistically significant analysis of variance by the physiological function (i.e., cell density (CD), chlorophyll a (Chl *a*), protein, malonaldehyde (MDA), superoxide dismutase (SOD), and carbonic anhydrase (CA)) of *C.weissflogii* under coexistence of salinity (Sal) and acidity (pH).

**Fig. 1** The map indication of the seawater origin.

**Fig. 2** The cultured growth cycle curve of *T. weissflogi*. Data are mean ± SD (*n*=3)

**Table 1** Correlations between physiological function (i.e., cell density (CD), chlorophyll a (Chl *a*), protein, malonaldehyde (MDA), superoxide dismutase (SOD), and carbonic anhydrase (CA)) under different salinity (Sal) and acidity (pH).

|  | *pH* | | | | | *Sal* | | | | |
| --- | --- | --- | --- | --- | --- | --- | --- | --- | --- | --- |
|  | CD | Chl *a* | Protein | MDA | SOD | CD | Chl *a* | Protein | MDA | SOD |
| Chl *a* | 0.445 |  |  |  |  | -0.950^*^ |  |  |  |  |
| Protein | 0.465 | 0.275 |  |  |  | -0.314 | 0.370 |  |  |  |
| MDA | 0.742 | 0.168 | 0.900 |  |  | -0.702 | 0.778 | 0.870 |  |  |
| SOD | -0.878 | -0.195 | -0.766 | -0.966^*^ |  | -0.845 | 0.643 | 0.312 | 0.521 |  |
| CA | -0.996^**^ | -0.510 | -0.511 | -0.757 | 0.879 | -0.966^*^ | 0.835 | 0.258 | 0.594 | 0.950^*^ |

^*^ Significant difference at 0.01 < *p* <0.05; ^**^ Extremely significant difference at *p* <0.01

**Table 2.**  Statistically significant analysis of variance by the physiological function (i.e., cell density (CD), chlorophyll a (Chl *a*), protein, malonaldehyde (MDA), superoxide dismutase (SOD), and carbonic anhydrase (CA)) of *C.weissflogii* under coexistence of salinity (Sal) and acidity (pH).

| Parameter | Source of variation | df | MS | *F* | *p* |
| --- | --- | --- | --- | --- | --- |
| CD | pH | 3 | 1.99 | 3.85▲ | 5.05×10^-2^ |
|  | Sal | 3 | 2.58 | 4.99▲ | 2.63×10^-2*^ |
|  | Error | 9 | 0.518 |  |  |
| Chl *a* | pH | 3 | 577 | 19.6▲ | 2.77×10^-4**^ |
|  | Sal | 3 | 97.2 | 3.30 | 7.17×10^-2^ |
|  | Error | 9 | 29.5 |  |  |
| Protein | pH | 3 | 1.18×10^3^ | 5.69▲ | 1.05×10^-3**^ |
|  | Sal | 3 | 635 | 3.07 | 8.34×10^-2^ |
|  | Error | 9 | 207 |  |  |
| MDA | pH | 3 | 0.662 | 13.7▲ | 1.83×10^-2*^ |
|  | Sal | 3 | 0.113 | 2.33 | 0.142 |
|  | Error | 9 | 4.83×10^-2*^ |  |  |
| SOD | pH | 3 | 26.83 | 3.65 | 5.71×10^-2^ |
|  | Sal | 3 | 1.45 | 0.198 | 0.895 |
|  | Error | 9 | 7.35 |  |  |
| CA | pH | 3 | 7.60 | 2.35 | 0.140 |
|  | Sal | 3 | 3.88 | 1.20 | 0.364 |
|  | Error | 9 | 3.23 |  |  |

^*^Significant difference at 0.01 < *p* <0.05; ^**^ Extremely significant difference at *p* <0.01.

▲ F>F*α*=3.86


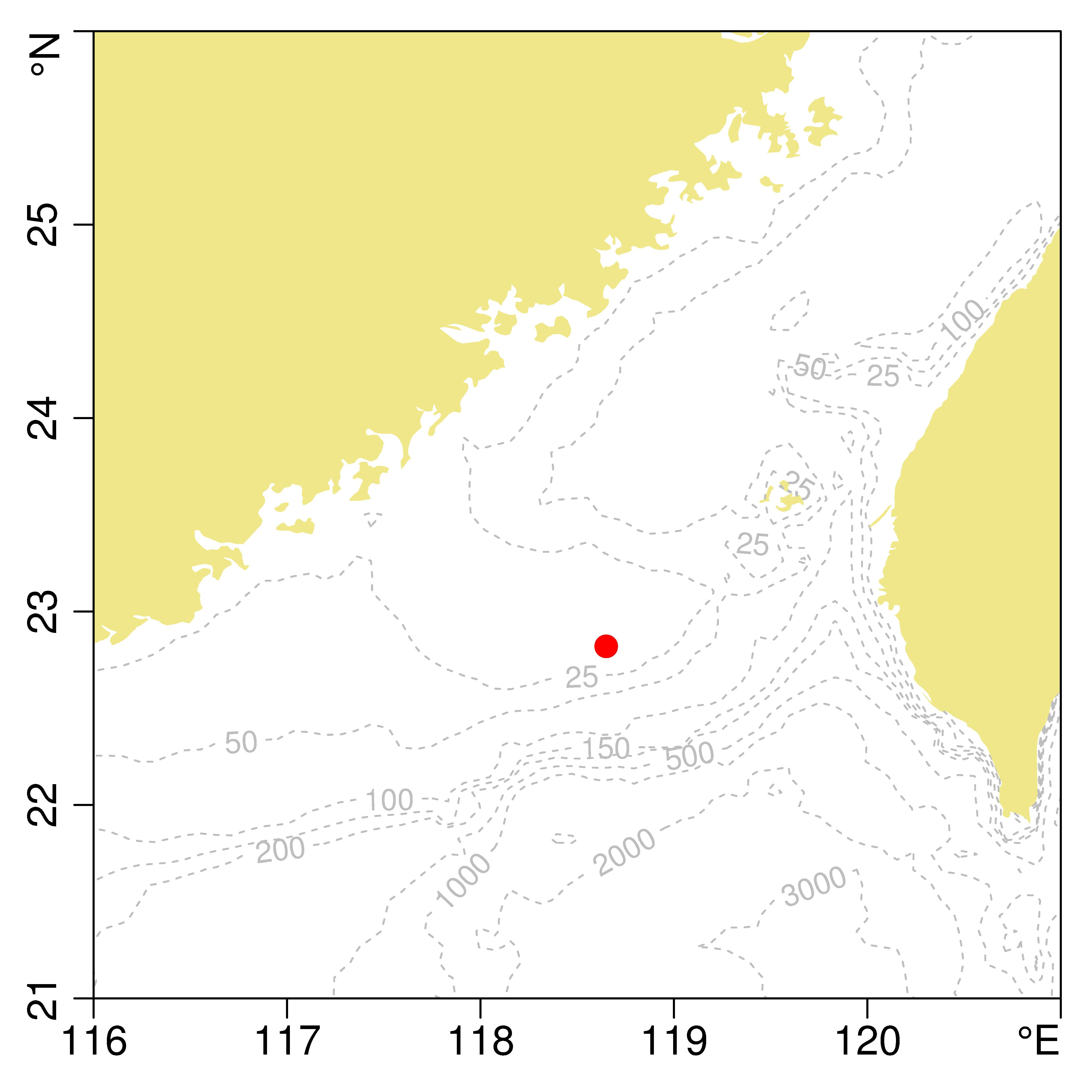


**Fig. 1** The map indication of the seawater origin (The R Programming Language, 3.0.3).

**Fig. 2** The cultured growth cycle curve of *T. weissflogi*. Data are mean ± SD (*n*=3)
